# Prevalence of Cardiomyopathy in Patients with Type 1 Diabetes Mellitus

**DOI:** 10.3390/jcm13185351

**Published:** 2024-09-10

**Authors:** Oscar Daniel Fabila-de la Cruz, Eduardo Salif Luna-Avila, María del Pilar Sotelo-González, Andrés D. Litardo-Mosquera, Oscar Orihuela, Aldo Ferreira-Hermosillo

**Affiliations:** 1 Facultad de Medicina, Universidad Nacional Autónoma de México, Mexico City 04510, Mexico; o.danielfab04@gmail.com (O.D.F.-d.l.C.); esalif@gmail.com (E.S.L.-A.); 2 Servicio de Endocrinología, Hospital de Especialidades “Dr. Bernardo Sepúlveda”, Centro Médico Nacional Siglo XXI, Instituto Mexicano del Seguro Social, Mexico City 06720, Mexico; pilar.sotelo.gonzalez@gmail.com; 3 Endocrinología, Hospital Clínica San Francisco, Guayaquil 090511, Ecuador; andresdavid4444@hotmail.com; 4 Servicio de Cardiología, Hospital de Especialidades “Dr. Bernardo Sepúlveda”, Centro Médico Nacional Siglo XXI, Instituto Mexicano del Seguro Social, Mexico City 06720, Mexico; orihuelao@yahoo.com.mx; 5 Unidad de Investigación Médica en Enfermedades Endocrinas, Centro Médico Nacional Siglo XXI, Instituto Mexicano del Seguro Social, Mexico City 06720, Mexico

**Keywords:** type 1 diabetes mellitus, left ventricular diastolic dysfunction, diabetic cardiomyopathy, echocardiography, cardiovascular diseases

## Abstract

**Background:** Diabetic cardiac muscle disease or diabetic cardiomyopathy (DbCM) comprises a set of myocardial lesions that are not associated with coronary atherosclerosis or high blood pressure. It is characterized by fibrosis and hypertrophy, which ultimately results in heart failure. Diastolic dysfunction (DD) has been shown to be the first manifestation of diabetic cardiomyopathy. Currently, there are few studies on the prevalence of diabetic cardiomyopathy in adult patients diagnosed with type 1 diabetes mellitus (T1D). **Methods:** The study included 75 adult participants who underwent an echocardiogram. Data on their comorbidities were collected from their medical records and biochemical parameters were analyzed in blood and urine samples. **Results:** We found that the prevalence of DbCM in our T1D population was more than one-third (34%), which exceeded the prevalence reported in studies with adolescents and that reported in the population without diabetes. Also, we found that the probability of developing DD after 20 years of T1D diagnosis was 78%. **Conclusions:** Recommendations need to be issued in relation to diabetic cardiomyopathy to carry out secondary prevention in adult patients with T1D. More multicenter studies, which include a larger population, from different regions of the world need to be performed.

## 1. Introduction

Type 1 diabetes mellitus (T1D) is an autoimmune metabolic disorder characterized by absolute insulin deficiency. As a consequence of this disorder, the main organs and systems affected are the heart and the circulatory system. The clinical manifestations associated with cardiovascular disease (CVD) represent the main cause of morbidity and mortality in patients with diabetes mellitus [1]. Much of this known association applies to patients with type 2 diabetes mellitus (T2D), so when adjustments are made for patients with T1D, the age-adjusted relative risk for cardiovascular complications is higher than that for patients with T2D and four to eight times that of the general population [2].

Myocardial lesions that are not related to coronary atherosclerosis or arterial hypertension are known as diabetic cardiac muscle disease or diabetic cardiomyopathy (DbCM) [3]. DbCM is characterized mainly by changes in the structure and function of the heart, typically due to fibrosis and hypertrophy, resulting in diastolic dysfunction [4]. In other words, diabetic cardiomyopathy is characterized by diastolic dysfunction (DD) of the left ventricle (LV). The main structural feature of DbCM is based on LV concentric remodeling, whereas the most common histopathological finding is fibrosis, which can be perivascular, interstitial, or both [5,6,7]. Several studies have shown that LV diastolic dysfunction (DD) is an early phase of DbCM, which precedes LV systolic dysfunction (SD) and clinical signs of heart failure (HF) [8,9,10,11]. In fact, SD of the LV, defined as an ejection fraction less than 45%, have been reported in 1–2% of patients with T1D. These structural changes in the hearts of patients with T1D could be related to the increased prevalence of cardiovascular diseases and its increased risk of mortality.

Among its basic functions, the microvasculature of the myocardium facilitates the supply of oxygen, nutrients and hormones, as well as the elimination of metabolic end products from the myocardium. In patients with diabetes mellitus, endothelial dysfunction is generated, altering these metabolic processes, but it is also known that there is an increase in the accumulation of triglycerides in the myocardium, which is associated with an increase in the mass of the left ventricle, leading to systolic and diastolic dysfunction [12,13]. These processes of myocardial damage occur earlier in patients with T1D, as insulin deficit promotes a reduction in glucose uptake in the myocardium. This decreases its oxidation and increases its dependence on free fatty acids as a source of energy, which promotes the production of reactive oxygen species (ROS), mitochondrial dysfunction, and calcium alteration, contributing to the development of cardiomyopathy [14].

In a cross-sectional study that included 1093 patients from the Steno Diabetes Center with a mean age of 49.6 years, Jensen et al. reported that 14.4% had diastolic dysfunction. In the same population, but analyzing patients without albuminuria, diastolic dysfunction was reported in 7.6% [10]. In an Italian cohort, Palmieri et al. also found a prevalence of diastolic dysfunction of 8% in patients with T1D with a mean age of 33 ± 10 years [11]. A Mexican study of a cohort of adolescents with a mean age of 14 ± 1.5 years reported a diastolic dysfunction prevalence of 18% [15]. The highest prevalence reported in T1D was found by Wai et. al. in an adult population with a mean age of 39 ± 14 years and median duration of diabetes mellitus of 21 years, which was 69% [16]. Literature data about this subject are scarce.

In Mexico and many parts of the world, studies of adult patients with T1D with CVD are insufficient. The present study aimed to determine the prevalence of ventricular diastolic dysfunction and its main associated factors in adult patients with T1D. The results obtained allowed us to obtain a representative value for the prevalence of cardiomyopathy in adult patients with T1D, as well as an understanding of the correlations between the risk factors that were identified and the probability of patients developing DD.

## 2. Materials and Methods

### 2.1. Study Population and Inclusion and Exclusion Criteria

Patients with T1D from the Diabetes Clinic of the Hospital de Especialidades of the Centro Médico Nacional Siglo XXI of the IMSS, Mexico City, Mexico who consecutively attended an assessment between February and December 2022 were invited to participate in the study. For those interested, we explained the study objective, and their informed signed consent was required. A day after that, a blood sample was obtained after 8 h fasting for performing biochemical tests. On this day, we programmed their echocardiogram according to the cardiologist agenda.

Patients with T1D as defined by the American Diabetes Association (ADA), with a glomerular filtration rate (GFR) greater than 15 mL/min and no associated neoplasms autoimmune diseases, such as lupus or rheumatoid arthritis, or a history of hospitalization during the last 3 months were included. Pregnant women or those who were hospitalized during the study period were excluded.

Considering an estimated prevalence of 18% in a finite population of 150 patients with T1D, which corresponds to the total number of patients seen in our clinic, a calculation of sample size for prevalence was performed, considering a level of confidence of 95% and a precision of 5%, yielding a requirement for 75 participants.

At the patients’ appointments, a blood sample and 24 h urine collection were obtained for assessing glycated hemoglobin (HbA1c), total cholesterol, high-density lipoprotein (HDL), low-density lipoprotein (LDL), creatinine, glucose, uric acid, and 24 h urine microalbuminuria. Clinical data on age, sex, duration of diabetes mellitus, basal insulin dose, insulin bolus dose, prevalence of hypertension, hypertriglyceridemia, hypercholesterolemia, hypothyroidism and the presence of microvascular complications were obtained. Weight and height were recorded to calculate the body mass index (BMI) and waist circumference. Based on these data, the GFR was calculated by means of the CKD-EPI 2021 formula.

### 2.2. Anthropometric Evaluation

We registered weight and height, and waist circumference (WC). Only one investigator performed anthropometric measurements using a calibrated scale with an integrated stadiometer. WC was evaluated at the middle point between the inferior rim of the last costal arch and the superior rim of the anterosuperior iliac spine. Body mass index (BMI) was calculated as weight divided by height squared (kg/m^2^). Blood pressure was determined after 10 min of rest, during a fasting state, without coffee or tobacco ingestion. The values were averaged after two different measurements with a 5 min difference between them using a calibrated sphygmomanometer.

### 2.3. Biochemical Evaluation

For biochemical analysis, we obtained a 6 mL sample in a BD Vacutainer (BD Franklin Lakes, Wayne, NJ, USA) and centrifuged it at 3150× *g* for 15 min. Then, serum was analyzed with a commercial kit for glucose, cholesterol, HDL, and triglycerides (COBAS 2010 Roche Diagnostics, Indianapolis, IN, USA) using photocolorimetry with spectrophotometry (2010 Roche Diagnostics, Indianapolis, IN, USA). HDL samples were treated with enzymes modified with polyethylene glycol and dextran sulfate and analyzed with the same photocolorimetric technique. HbA1c was evaluated by turbidimetric immunoanalysis (COBAS 2010 Roche Diagnostics, Indianapolis, IN, USA). LDL was calculated with the Friedewald formula if triglycerides were <400 mg/dL.

### 2.4. Echocardiogram Assessment

The participants were sent for an echocardiogram evaluation by the cardiology service. Echocardiography studies were performed with iE33^®^ equipment (Philips Medical System, Andover, MA, USA). The images were obtained in 2D and M mode to approach the parasternal and apical regions with the patient in the lateral decubitus position. With respect to the measurements, the recommendations of the American Society of Echocardiography [17] were followed and were performed by a single specialist.

For the analysis of the left ventricular mass, the Deveraux formula was used, defining left ventricular hypertrophy as a mass greater than 95 g/m^2^ in females and more than 115 g/m^2^ in males. Regarding the assessment of left ventricular function, according to the recommendations of the American Society of Echocardiography published in 2016 [17], left ventricular dysfunction was considered if at least 3 of the following criteria were met: average E/e ratio > 14, septal velocity e′ < 7 cm/s or lateral velocity e′ < 10 cm/s, tricuspid regurgitation velocity > 2.8 m/s, and left ventricular volume index > 34 mL/m^2^.

### 2.5. Statistical Analysis

The Shapiro–Wilk test was used to establish the normality of the distribution of the quantitative variables, which are described as the means ± standard deviations or medians with interquartile ranges if not normally distributed. Qualitative variables are reported as frequencies or percentages. The chi-squared test or Fisher’s exact test was used to search for associations between qualitative variables, depending on the numbers in each cell in the 2 × 2 contingency table (fewer or more than 5), and the Mann–Whitney U test or *t* test was used for quantitative variables depending on its distribution. To evaluate the time of diagnosis of T1D at which there is a greater probability of developing diastolic dysfunction, a receiver operating characteristic (ROC) curve was used. The cutoff point was selected via the Youden index (sensitivity + specificity − 1). *p* < 0.05 was considered statistically significant. The data were analyzed with the statistical software SPSS 23.0.

## 3. Results

### Prevalence of Diabetic Cardiomyopathy in the Population Studied

**Figure 1:**
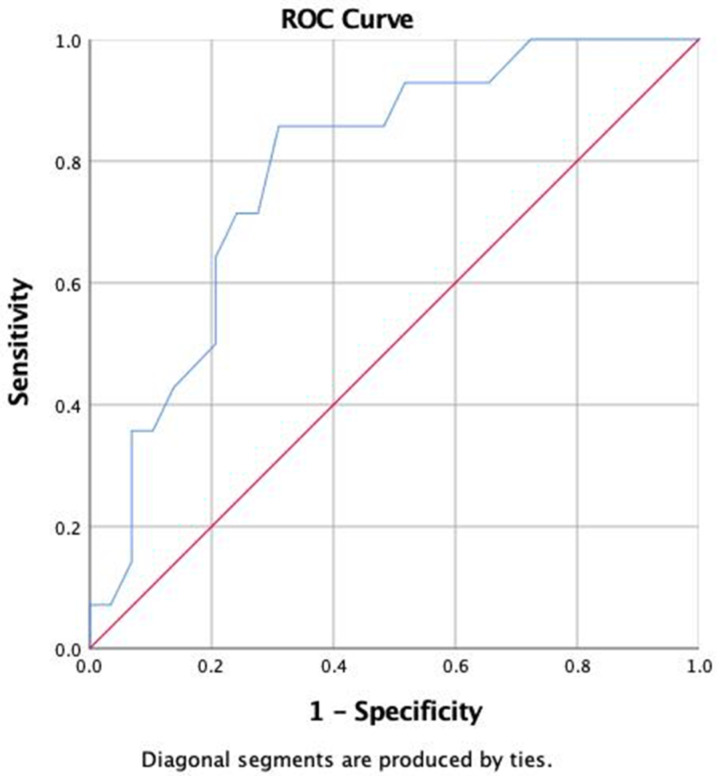
ROC curve of years since diagnosis of T1D for the development of diastolic dysfunction.

A sample of 75 patients with T1D was obtained. Of these, 70% were female, with a mean age of 35 ± 11 years, 30% were male, with an age of 35 ± 13 years, 18% had hypertension, 47% had hypercholesterolemia, 27% had hypertriglyceridemia, 25% had diabetic kidney disease (DKD), 23% had diabetic neuropathy (DN), and 24% had retinopathy. The incidence of DD was 34%, with 87% of patient having stage I disease and 13% having stage II, with an E/A of 1.2 (0.41) cm/s and an ejection fraction of 65 ± 5%. The average BMI was 25.2 ± 3.9 kg/m^2^, and the median HbA1c was 8.8% (7.6–9.8%). Patients with DD had a longer duration of diabetes mellitus, a higher uric acid concentration, a lower E/A ratio, and a higher incidence of retinopathy and kidney disease. There were no differences in treatment for hypertension, dyslipidemia, kidney disease, or other oral treatments for diabetes mellitus between the groups (Table 1). On ROC curve analysis, we found that time since diagnosis of T1D allowed detection of those with DD (area under the curve (AUC) 78%, 95% CI 65–93%). A cutoff point of 20 years showed sensitivity of 86%, specificity of 69%, positive likelihood ratio (LR+) of 2.77, and negative likelihood ratio (LR-) of 0.20 (Figure 1 and Supplementary Table S1).

## 4. Discussion

The main characteristic of DbCM is DD, but despite this manifestation being known as one of the first cardiovascular complications that can be detected in people living with T1D, there are no concrete data that allow us to reach a consensus about the worldwide prevalence of DbCM and specifically of DD in this population.

In our study, we observed a prevalence of DD of 34% in patients with a mean age of 35 years 27 ± 9 years after diagnosis of T1D. This differs from that reported by Wai et al. in a population of 136 people with T1D from Australia. They found a prevalence of abnormalities of 29% in the echocardiograms performed, in which 69% showed DD, with an average patient age of 39 years and a median T1D duration of 21 years (IQR 11–29 years) [16]. However, our prevalence is similar to the 37.1% reported by Fernández-Fúnez et al. in 35 patients with T1D evaluated in Spain with a mean age of 27.8 ± 7.5 years and a mean time of T1D disease progression of approximately 11.1 ± 7.1 years. Importantly, this study included a healthy control population in which no cases of DD were found [18]. As observed, it seems that the prevalence of DD could be related to the time since diagnosis of diabetes mellitus, as well as the studied population.

According to our results, the variables that proved to be most important for the presence of DD were the T1D progression time, uric acid concentrations, and the presence of diabetic nephropathy and diabetic retinopathy. In several studies in patients with T1D, age is the strongest predictor of alterations on echocardiography, with an increase in risk of 46% with each year of increase in age after 40 years [10,16,19]. However, other studies have not reported an important association between clinical variables and abnormal echocardiogram results [16,18]. In our study, a probability of up to 78% of patients developing DD was observed 20 years after the diagnosis of T1D, with a sensitivity and specificity of 86% and 69%, respectively. In accordance with this result, we suggest conducting a screening study with echocardiography in patients living with T1D after this age to establish a timely diagnosis and be able to offer adequate measures in these patients.

The relationship between the presence of DD, age, anthropometric values such as waist circumference, BMI, and systolic and diastolic blood pressure, and the control of metabolic aspects such as HbA1c, lipid metabolism, and microalbuminuria were not significant in our study. This finding contrasts with that of Jensen et al. in the Thousand & 1 Study, where clinical characteristics such as age (OR 2.1, 1.8–2.4), diabetes mellitus duration (more than 10 years) (OR 1.7, 1.4–1.9), systolic and diastolic blood pressure (OR 2.7, 1.9–3.8 and OR 1.8, 1.0–3.1, respectively), estimated glomerular filtration rate (OR 3.8, 2.5–5.9), micro and macroalbuminuria (OR 2.0, 1.3–3.0 and OR 5.9, 3.8–9.3, respectively), and the presence of proliferative retinopathy (OR 3.6, 2.3–5.8) and DN (OR 3.8, 2.7–5.3) were associated with abnormal myocardial function, particularly the presence of macroalbuminuria (OR 5.2, 2.9–10.13), age (OR 2.1, 1.7–2.5), and female sex (OR 1.9, 1.2–2.8), which remained significant on multivariate analysis [10]. Wai et al. also reported that age (OR 9.40, 2.68–33.04) and increased BMI (OR 1.17, 1.01–1.36) are independent predictors of an abnormal echocardiogram [16]. Guglielmi et al. reported that microalbuminuria is associated with a more severe impairment of cardiac diastolic function [20]. In our study, only previous diagnosis of DKD and diabetic retinopathy were higher in the group with DD. Together, these complications have been related to each other and could be related to the higher exposure to glycemic alterations for a long period, which correlates with our finding that time since diabetes mellitus diagnosis is related to the presence of DD. Finally, we observed that patients with DD had higher concentrations of uric acid, a well-known proinflammatory marker. Unfortunately, we could not perform other inflammation markers such as cytokines that could help us to corroborate this finding.

Traditionally, HbA1c is considered the most powerful risk factor of cardiovascular disease in T1D patients, whereas other CVD risk factors, such as blood pressure or LDL cholesterol, have a demonstrable independent contribution only after 15 to 20 years of diagnosis. We consider that the lack of association with glycemic control assessed by HbA1c is due to the fact that a single measurement does not reflect long-term control of diabetes mellitus. Although other studies have correlated changes in ventricular filling and LV pressure rate with significant improvements in HbA1c, especially in populations with less exposure to the disease, no remission of DD has been reported, even in those with adequate glycemic control or statin treatment. Furthermore, Gul et al. evaluated 81 patients with T1D and 51 healthy volunteers using tissue Doppler imaging (TDI) to evaluate the effects of diabetes mellitus in DD. As in our study, they observed that HbA1c was not correlated with TDI parameters, in contrast to diabetes mellitus duration [9]. Evaluating the role of glycemic variability in the development of DbCM with continuous glucose monitors and the evaluation of HbA1c variations over time would be relevant. To the best of our knowledge, there are no studies on this topic currently.

The prevalence of DD is higher in the population with T2D. In the United States, Boyer et al. studied 61 patients without hypertension with a mean age of 45.6 ± 8 years and a mean progression time of 5.8 ± 5.5 years. In this population, they found that the prevalence of DD diagnosed by conventional echocardiography was 46% and that this increased to 74% when DD was evaluated by tissue Doppler imaging [21]. On the other hand, in a more recent systematic review and meta-analysis that included 27 studies and 2959 patients with T2D, Bouthoorn et al. reported a prevalence of DD of 35% (24–46%) for non-hospitalized patients and up to 48% (38–59%) for hospitalized patients. However, there was great heterogeneity in the studies analyzed, and this is attributed to the lack of a standardized definition of DD, differences in the populations studied, differences in ages, and differences in the durations of T2D progression [22]. In Mexico, Sánchez-Barriga et al. evaluated 61 patients with T2D and reported that the prevalence of DD was 58%. The average age of the patients with DD was 54 ± 8 years, with a diabetes mellitus progression time of 5.3 ± 2.6 years. In the comparison of patients with and without DD, significant differences in concentrations of HbA1c (12.0 ± 1.9% vs. 8.0 ± 1.5%, *p* < 0.001), triglycerides (280 ± 89 mg/dL vs. 195 ± 54 mg/dL, *p* < 0.001) and cholesterol (224 ± 61 mg/dL vs. 186 ± 27 mg/dL, *p* = 0.001) were detected, but there were no differences in age or disease progression time [23]. As mentioned, these parameters were not significantly different between our patients with T1D with and without DD, which suggests that the cardiovascular complications related to T1D and T2D have different times and ages at presentation, as well as different associated factors. This could be related to the longer exposure of T1D patients to hyperglycemia, as well as to the proinflammatory and secondary pro-oxidant state, because the disease occurs at a younger age.

One of the main limitations of our study is that the sample is small and from a single diabetes care center in a highly specialized tertiary level unit, which has the necessary resources to carry out studies such as echocardiography; however, our sample is significant since the calculated sample size was reached within a finite population with 150 patients in total. In addition, many of the patients included are not residents of Mexico City, since the hospital provides medical care to patients from the central and southern regions of the country. However, we consider it important that DD in patients with T1D be evaluated in other T1D care centers in our country and in other countries to obtain more information on the real prevalence of this condition. Another limitation is that we were unable to assess other potential causes of DD, such as genetic factors or inflammation markers. Those should be considered in future studies. However, the genetic drivers of left ventricular diastolic dysfunction are not defined, and it seems that the genetic predisposition to increase in BMI and altered glucose homeostasis are indirectly related to its development [24]. Finally, another limitation of our study lies in its cross-sectional nature, which limits the ability to find causality for the development of DD. This requires a longitudinal approach, with a larger sample in a more heterogeneous population.

Among the main strengths of our study, patients living with T1D are under regular follow-up at the Diabetes Clinic, which in the long term will allow us to understand their evolution and compare the results with those of other series of patients whose characteristics differ from those of our population. Similarly, another strength lies in the fact that our study did not present interobserver variability for the diagnosis on the basis of echocardiography, so it was not necessary to apply evaluation methods on the basis of the concordance in the interpretation of the results.

The use of echocardiography for DbCM screening in patients with T1D is currently not well standardized and is only considered when other cardiovascular causes are present. However, some studies have suggested its utility in a broader spectrum. In a study conducted by Weber et al., they aimed to study cardiac changes in asymptomatic and normotensive patients with TD1 compared with a control group using conventional two-dimensional Doppler and advanced speckle tracking echocardiography. Patients with T1D had an average age of 32.7 ± 8.5 years and an average time since diabetes diagnosis of 15 ± 9.1 years. They observed a decrease in diastolic function in comparison to the control group, but proper DD could not be diagnosed. They concluded that even with preserved systolic function and subtle variations in diastolic function, there could be a subtle presence of DD in patients with T1D [25]. In a study by Jensen et al. on 1093 patients with T1D, they sought to use echocardiography as a prognostic tool for cardiovascular disease. The patients were aged 39.2–60.3 years with an average time since diabetes diagnosis of 25.8 years. They concluded that its use significantly increases the identification of myocardial dysfunction in patients with T1D without known heart disease [26].

The results obtained from our study, in comparison with those of other studies carried out in patients with T1D, demonstrate the usefulness and importance of performing echocardiographic studies for the early detection of DbCM and specifically DD, which is one of the first manifestations of DbCM. We believe that clinical trials are necessary to establish secondary prevention measures related to attenuating the cardiovascular complications of T1D, even considering the use of other drugs that have demonstrated cardiovascular benefits in patients with T2D, such as SGLT2 inhibitors or GLP-1 analogues.

## 5. Conclusions

The prevalence of DD found in our study population was greater than that reported in other case series and is similar to that reported in studies with adult populations, so it is important not to underestimate the prevalence of diabetic cardiomyopathy in adult patients with T1D. Among the most relevant risk factors, time since progression since the diagnosis of DD is an important factor for screening and preventing cardiomyopathy secondary to diabetes mellitus. With respect to biochemical markers, uric acid, an inflammatory marker, was significantly different between the groups with and without DD, so a subsequent study of inflammatory markers could help us clarify the microenvironment in which DD occurs. In the groups with and without DD, there was a difference in microvascular complications, the prevalence of which was greater in the group with DD, and this was also associated with the number of years since diabetes diagnosis. Finally, with respect to glycemic control, it is necessary to study a larger population to perform an analysis of HbA1c and establish an association.

## Figures and Tables

**Table 1:** Comparison of clinical and biochemical variables between patients with type 1 diabetes with and without diastolic dysfunction.

Variable	With DD (n = 26)	Without DD (n = 49)	*p*
Age (years)	40 ± 14	35 ± 11	0.21
Sex, women	73%	76%	1.00
BMI (kg/m^2^)	25.9 ± 4.12	25.2 ± 4.3	0.58
WC (cm)	88 ± 9	89 ± 11	0.85
Systolic blood pressure (mmHg)	112 + 16	114 +17	0.74
Diastolic blood pressure (mmHg)	72 + 10	71 + 9	0.66
eGFR (mL/min/1.73 m^2^)	97 ± 22	96 ± 22	0.90
Diabetes mellitus duration (years)	27 ± 9	16 ± 10	0.002
Basal dose (IU/day)	40 ± 22	49 ± 19	0.21
Bolus dose (IU/day)	16 ± 10	16 ± 6	0.96
HbA1c (%)	9.2 (7.6–9.9)	9 (7.9–9.7)	0.77
Total cholesterol (mg/dL)	170 ± 32	160 ± 29	0.35
HDL (mg/dL)	45 (38–53)	48 (40–56)	0.83
LDL (mg/dL)	97 ± 25	86 ± 25	0.18
Creatinine (mg/dL)	0.89 (0.77–1.06)	0.81 (0.77–0.95)	0.35
Glucose (mg/dL)	151 ± 78	155 ± 74	0.85
Uric acid (mg/dL)	5.70 ± 1.3	3.96 ± 1.6	0.05
24 h urine microalbuminuria (µg/d)	37 (13–48)	16 (9–54)	0.36
E (m/s)	83 ± 6	81 ± 18	0.73
A (m/s)	62 ± 0.7	57 ± 22	0.52
IVRT (ms)	86.5 ± 7.7	85.8 ± 18.5	0.93
E/A (cm/s)	0.94 ± 0.48	1.34 ± 0.28	0.02
LVEF (%)	66 ± 5	64 ± 5	0.10
Hypertension	27%	17%	0.46
Hypertriglyceridemia	33%	29%	0.74
Hypercholesterolemia	54%	43%	0.51
Hypothyroidism	36%	62	0.19
Diabetic kidney disease (DKD)	46%	14%	0.046
Diabetic neuropathy (DN)	20%	14%	0.67
Diabetic retinopathy (DR)	33%	3%	0.013

Quantitative data are expressed as means ± standard deviations or medians (interquartile ranges). Qualitative data are expressed as percentages. *p* values were obtained via chi-squared test for associations between qualitative variables and the Mann–Whitney U test or *t* test for quantitative variables. *p* < 0.05 was considered statistically significant. Abbreviations: BMI, body mass index; WC, waist circumference; eGFR, estimated glomerular filtration rate; HDL, high-density lipoprotein; LDL, low-density lipoprotein; E, early diastolic flow peak velocity of the mitral valve; A, peak velocity of late left ventricular filling; IVRT, isovolumic relaxation time; E/A, E/A ratio; LVEF, left ventricular ejection fraction.

## Data Availability

The data analyzed in the study are available from the corresponding author upon reasonable request.

## Supplementary Materials

The following supporting information can be downloaded at https://www.mdpi.com/article/10.3390/jcm13185351/s1. Table S1: Contingency table of the cut-off years since diagnosis and the presence of DD detected with echocardiogram.

## References

[1] Rao Kondapally Seshasai S., Kaptoge S., Thompson A., Di Angelantonio E., Gao P., Sarwar N., Whincup P.H., Mukamal K.J., Gillum R.F., Holme I. (2011). Diabetes Mellitus, Fasting Glucose, and Risk of Cause-Specific Death. N. Engl. J. Med..

[2] Donlo I.C., Donlo M.G. (2013). Enfermedad cardiovascular en la diabetes mellitus tipo 1. Más que mera coincidencia. Med. Clín..

[3] Cosson S., Kevorkian J.P. (2003). Left ventricular diastolic dysfunction: An early sign of diabetic cardiomyopathy?. Diabetes Metab..

[4] Ritchie R.H., Zerenturk E.J., Prakoso D., Calkin A.C. (2017). Lipid metabolism and its implications for type 1 diabetes-associated cardiomyopathy. J. Mol. Endocrinol..

[5] Theilade S., Rossing P., Jensen J.S., Jensen M.T. (2017). Arterial-ventricular coupling in type 1 diabetes: Arterial stiffness is associated with impaired global longitudinal strain in type 1 diabetes patients—The Thousand & 1 Study. Acta Diabetol..

[6] Di Cori A., Di Bello V., Miccoli R., Talini E., Palagi C., Donne M.G.D., Penno G., Nardi C., Bianchi C., Mariani M. (2007). Left ventricular function in normotensive young adults with Well-Controlled Type 1 Diabetes Mellitus. Am. J. Cardiol..

[7] Levelt E., Gulsin G., Neubauer S., McCann G.P. (2018). MECHANISMS IN ENDOCRINOLOGY: Diabetic cardiomyopathy: Pathophysiology and potential metabolic interventions state of the art review. Eur. J. Endocrinol..

[8] Jensen M.T., Sogaard P., Andersen H.U., Bech J., Hansen T.F., Biering-Sørensen T., Jørgensen P.G., Galatius S., Madsen J.K., Rossing P. (2015). Global longitudinal strain is not impaired in Type 1 diabetes patients without albuminuria. JACC Cardiovasc. Imaging.

[9] Gul K., Celebi A.S., Kacmaz F., Ozcan O.C., Ustun I., Berker D., Aydin Y., Delibasi T., Guler S., Barazi A.O. (2009). Tissue Doppler imaging must be performed to detect early left ventricular dysfunction in patients with type 1 diabetes mellitus. Eur. J. Echocardiogr..

[10] Jensen M.T., Sogaard P., Andersen H.U., Bech J., Hansen T.F., Galatius S., Jørgensen P.G., Biering-Sørensen T., Møgelvang R., Rossing P. (2014). Prevalence of systolic and diastolic dysfunction in patients with type 1 diabetes without known heart disease: The Thousand & 1 Study. Diabetologia.

[11] Palmieri V., Capaldo B., Russo C., Iaccarino M., Pezzullo S., Quintavalle G., Di Minno G., Riccardi G., Celentano A. (2008). Uncomplicated type 1 diabetes and preclinical left ventricular myocardial dysfunction: Insights from echocardiography and exercise cardiac performance evaluation. Diabetes Res. Clin. Pract..

[12] Szczepaniak L.S., Dobbins R.L., Metzger G.J., Sartoni-D’Ambrosia G., Arbique D., Vongpatanasin W., Unger R., Victor R.G. (2003). Myocardial triglycerides and systolic function in humans: In vivo evaluation by localized proton spectroscopy and cardiac imaging. Magn. Reson. Med..

[13] Rijzewijk L.J., Van Der Meer R.W., Smit J.W.A., Diamant M., Bax J.J., Hammer S., Romijn J.A., De Roos A., Lamb H.J. (2008). Myocardial steatosis is an independent predictor of diastolic dysfunction in type 2 diabetes mellitus. J. Am. Coll. Cardiol..

[14] Horton W.B., Barrett E.J. (2020). Microvascular dysfunction in diabetes mellitus and cardiometabolic disease. Endocr. Rev..

[15] Herrera-Márquez R., Peralta-Cortázar C., Contreras-Rodríguez A., Hernández-Rodríguez J., Manjarrez-Gutiérrez G. Subclinical Left Ventricular Diastolic Dysfunction in Adolescents with Type 1 Diabetes. Boletín Médico Del Hospital Infantil De México (English Edition). https://www.elsevier.es/en-revista-boletin-medico-del-hospital-infantil-201-articulo-subclinical-left-ventricular-diastolic-dysfunction-X2444340914395327.

[16] Wai B., Patel S.K., Ord M., MacIsaac R.J., Jerums G., Srivastava P.M., Burrell L.M. (2014). Prevalence, predictors and evolution of echocardiographically defined cardiac abnormalities in adults with type 1 diabetes: An observational cohort study. J. Diabetes Complicat..

[17] Nagueh S.F., Smiseth O.A., Appleton C.P., Byrd B.F., Dokainish H., Edvardsen T., Flachskampf F.A., Gillebert T.C., Klein A.L., Lancellotti P. (2016). Recommendations for the Evaluation of Left Ventricular Diastolic Function by Echocardiography: An Update from the American Society of Echocardiography and the European Association of Cardiovascular Imaging. J. Am. Soc. Echocardiogr..

[18] Fernández-Fúnez Á., Cabrera R., Hernández A., Requejo R., Rueda A., Fernández-Zamora F., Beato J.L. (2000). Insuficiencia cardíaca/trasplante cardíaco. Rev. Esp. Cardiol..

[19] Andersen N.H., Hansen T.K., Christiansen J.S. (2007). Changes in glycaemic control are related to the systolic function in type 1 diabetes mellitus. Scand. Cardiovasc. J..

[20] Guglielmi M.D., Pierdomenico S.D., Salvatore L., Romano F., Tascione E., Pupillo M., Porreca E., Imbastaro T., Cuccurullo F., Mezzetti A. (1995). Impaired Left Ventricular Diastolic Function and Vascular Postischemic Vasodilation Associated with Microalbuminuria in IDDM Patients. Diabetes Care.

[21] Boyer J.K., Thanigaraj S., Schechtman K.B., Pérez J.E. (2004). Prevalence of ventricular diastolic dysfunction in asymptomatic, normotensive patients with diabetes mellitus. Am. J. Cardiol..

[22] Bouthoorn S., Valstar G.B., Gohar A., Ruijter H.M.D., Reitsma H.B., Hoes A.W., Rutten F.H. (2018). The prevalence of left ventricular diastolic dysfunction and heart failure with preserved ejection fraction in men and women with type 2 diabetes: A systematic review and meta-analysis. Diab Vasc. Dis. Res..

[23] Sánchez-Barriga J.J., Rangel A., Castañeda R., Flores D., Frati A.C., Ramos M.A., Amato D. (2001). Left Ventricular Diastolic Dysfunction Secondary to Hyperglycemia in Patients with Type II Diabetes. Arch. Med. Res..

[24] Vaitinadin N.S., Shi M., Shaffer C.M., Farber-Eger E., Lowery B.D., Agrawal V., Gupta D.K., Roden D.M., Wells Q.S., Mosley J.D. (2022). Genetic determinants of body mass index and fasting glucose are mediators of Grade 1 diastolic dysfunction. J. Am. Heart Assoc..

[25] Weber T.R., Da Silva R.L., Cossul S., Alves M.S.L., Van Der Sander Lee S., Marques J.L.B. (2021). Echocardiographic evaluation in type 1 diabetes mellitus. Rev. Port. Cardiol..

[26] Jensen M.T., Sogaard P., Gustafsson I., Bech J., Hansen T.F., Almdal T., Theilade S., Biering-Sørensen T., Jørgensen P.G., Galatius S. (2019). Echocardiography improves prediction of major adverse cardiovascular events in a population with type 1 diabetes and without known heart disease: The Thousand & 1 Study. Diabetologia.

