# Supplementary material for: Prevalence of Cardiomyopathy in Patients with Type 1 Diabetes Mellitus
Source: J Clin Med. 2024 Sep 10;13(18):5351. doi: 10.3390/jcm13185351 (PMC11432088; doi:10.3390/jcm13185351)
Supplement: Supplementary file 1 [file jcm-13-05351-s001.zip › jcm-3172599-supplementary.pdf]

Supplementary Table S1. Contingency table of the cut-off years since diagnosis and the presence of DD detected with echocardiogram.

		Echocardiogram		
		Presence of DD (n)	Absence of DD (n)	Total (n)
Years since diagnosis	More than 20 years (n)	22	15	37
	Less than 20 years (n)	4	34	38
	Total (n)	26	49	

LVDD: Left ventricular diastolic disfunction
